# Comparison of Removal Behavior of Two Biotrickling Filters under Transient Condition and Effect of pH on the Bacterial Communities

**DOI:** 10.1371/journal.pone.0155593

**Published:** 2016-05-19

**Authors:** Xiang Tu, Jianjun Li, Rongfang Feng, Guoping Sun, Jun Guo

**Affiliations:** 1 School of Bioscience and Bioengineering, South China University of Technology, Guangzhou, 510006, PR China; 2 State Key Laboratory of Applied Microbiology Southern China, Guangzhou, 510070, PR China; 3 Guangdong Provincial Key Laboratory of Microbial Culture Collection and Application, Guangzhou, 510070, PR China; 4 Guangdong Institute of Microbiology, Guangzhou, 510070, PR China; CAS, CHINA

## Abstract

Although biotrickling filters (BTFs) applied under acidic condition to remove H_2_S from waste gases have been reported, the removal behavior of the acidic BTF under transient condition which was normal in most industry processes, and corresponding bacterial community have not been thoroughly studied. In the present study, two BTFs were run under neutral (BTFn) and acidic (BTFa) conditions, respectively. The results revealed that the removal performance of BTFa under transient condition was superior to that of BTFn; the maximum H_2_S eliminating capacities (ECs) achieved by BTFa and BTFn were 489.9 g/m^3^ h and 443.6 g/m^3^ h, respectively. High-throughput sequencing suggested that pH was the critical factor and several other factors including nutrient and the inlet loadings also had roles in shaping bacterial community structure. *Acidithiobacillus* was the most abundant bacterial group. The results indicated that BTF acclimation under acidic condition may facilitate generating microbial community with high H_2_S-degrading capability.

## Introduction

Hydrogen sulfide is the most prevalent odor compound generated from various industrial facilities such as municipal wastewater treatment plants, landfills, livestock farms and biogas plants. Under anaerobic conditions, the use of sulfate as the terminal electron acceptor by sulfate-reducing bacteria results in H_2_S production [1]. H_2_S emission is often problematic due to its offensive odor, toxicity and corrosion of equipment [2,3]. Several categories of H_2_S abatement methods have been developed over the past decades including physical, chemical and biological methods [4]. Biofiltration is among the most economical and environmental-friendly method, and its effectiveness in treating waste gases containing reduced sulfur compounds has been widely reported [5,6]. In biotrickling filters (BTFs), diverse microorganisms are immobilized onto the surface of the synthetic material, and form a thin layer of biofilm where pollutants are converted to harmless products. Microorganisms that can oxidize the reduced sulfur compounds are phylogenetically diverse, including members of the domain *Bacteria* and *Archaea* [7,8]. Because sulfuric acid is the major metabolic final product of H_2_S bio-oxidation, the pH in the filter bed tends to decrease sharply to a very low value [9]. Although H_2_S removal has been successfully achieved using bioreactors over a broad pH range [4,10], the use of acidic BTFs has several advantages [11,12]. For example, no alkali is needed to neutralize acid, and the acidity can inhibit the growth of many types of microorganisms that are not tolerant to acidy, thereby minimizing the clogging problems that are typically observed in neutral systems.

Many research works have been conducted to optimize the operating parameters, or to test the efficiency of filter materials and functional bacterial strains [13–15]. However, the information concerning the composition of microbial community was still limited. pH has been reported as a key factor that influences not only H_2_S removal but also the composition of the microbial community inside the biofilm [16]. It was reported that pH transition from neutral to acidic conditions drastically reduced the microbial diversity. Microorganisms belongs to *Acidithiobacillus* genera was found to be a major sulfur-oxidizing group in acidic bio-reactor [17–20]. BTFs are packed with inert materials over which the nutrients solution is trickled. When the nutrient solution flows downward, a pH gradient forms along the filter bed [21]. Moreover, the most significant elimination of H_2_S mainly occurred near the gas inlet section of the filter bed [22]. This phenomenon would result in an obvious vertical stratification of the nutrient or the metabolites along the filter bed. Together with these differences, it is speculated that the spatial distribution of the structure of the microbial communities may be different but correlated with various environmental factors. In recent years, the performances of the bioreactors treating H_2_S-containing waste gases under steady conditions has been extensively evaluated [23–25]. But in fact, the most off-gases generated at industrial sites are characterized by the fluctuation of the inlet loading rates caused by sudden increases of the influent concentration, which usually varied diurnally or hourly by several orders of magnitude [26]. The extent of the negative effect of the fluctuation on the performance of the BTF probably depends on the microbial composition [27,28]. However, what are driving forces shaping the microbial community during the H_2_S biofiltration? And to what extent do the environmental factors affect the microbial communities?

Based on the above considerations, in the present work, two BTFs were initially acclimated under steady condition at pH 7.0 and pH 4.0, respectively. The capabilities of both BTFs tolerant to the loading rates shock in short times were firstly evaluated. The composition and the structure of the bacterial communities within the two BTFs were also analyzed by a high throughput sequencing technique in attempt to find the main factors affecting the formation of the bacterial communities, and to reveal the interactive relationship between the bacterial composition and the environmental factors including pH, nutrient or inlet loading rates.

## Materials and Methods

### Biotrickling filters setup and its operation

**Fig 1:**
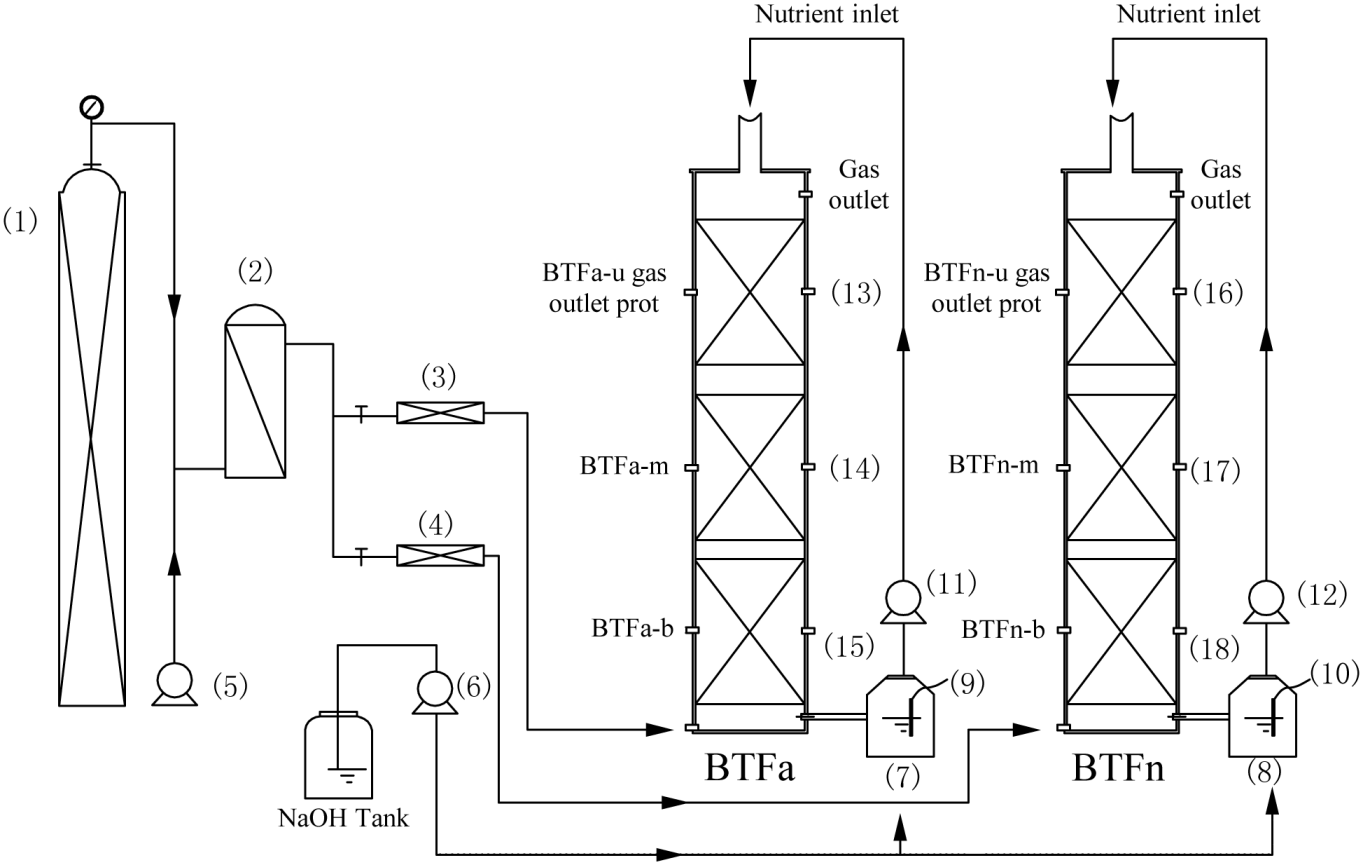
Schematic diagram of the biotrickling filter. (1) H_2_S cylinder, (2) Mixing chamber, (3,4) Gas flowmeter, (5) Air compressor, (6) NaOH dosing pump, (7,8) Nutrient tank, (9,10) pH probe, (11,12) Peristaltic pump, (13–15) BTFa-u/m/b port for sampling packing materials, (16–18) BTFn-u/m/b port for sampling packing materials.

Two identical bench scale BTFs (Fig 1) constructed with plexiglass column were used in the present study. Each BTF had a total height of 82 cm and an inner diameter of 8 cm. The height of the packing bed was 60 cm, corresponding to an effective packed volume of 3.01 L. Volcanic stone was used as the packing material. This packing material featured particle sizes of 0.5–1.0 cm, 70% porosity and a bulk density of 0.69 g/cm^3^. A perforated sieve plate at the bottom of the column was used to support the packing material. Synthetic polluted gases were generated by mixing H_2_S vapors with a fresh airstream in a mixing chamber. The desired H_2_S inlet loading rates were obtained by adjusting the flow rate of either the H_2_S airstream or the fresh airstream. The BTFs were operated in the up-flow configuration. Along the column wall of each BTF were three ports for sampling packing materials in the upper, middle and bottom layers (BTFa-u, BTFa-m and BTFa-b for BTFa, BTFn-u, BTFn-m and BTFn-b for BTFn, respectively). Each sample was collected in triplicate using sterile tweezers, and transported immediately to the laboratory. Three ports located at opposite sides of the column and the inlet and outlet port were used for gas sampling to evaluate the H_2_S removal performance of each layer. Nutrient solution containing inorganic salt was stored in a continuously stirred, triangular, 2 L flask, and was continuously supplied to the BTFs using a peristaltic pump (BT300-YZ1515X, ShenChen, China) at a flow rate of 80 mL/min. The pH values of the nutrient solution were adjusted to 4.0 for BTFa and 7.0 for BTFn by automatically additing alkaline in real time using a dosing pump (SKEO, Italy). The nutrient solution was refreshed once a week to ensure sufficient nutrients and moisture for the growth of microorganisms inside the bed, as well as to avoid inhibition resulting from metabolic product.

During the acclimation period, two BTFs operated under steady condition for a month with a constant inlet concentration of H_2_S. After that to assess the effect of loading-rates shock on the performances of the two BTFs, batch experiments were performed in a transient condition at the empty bed retention times (EBRT) of 60 s, 30 s and 15 s, respectively. At each EBRT, the inlet concentrations of H_2_S gradually increased within 14 h. The ranges for H_2_S concentration at 60 s, 30 s and 15 s were set at 175–5858 mg/m^3^, 169–5028 mg/m^3^ and 69–1029 mg/m^3^, respectively.

### Microorganism preparation

To obtain microorganisms that can adapt to various pH environments, both BTFs were simultaneously inoculated with an acclimated microbial consortium and activated sludge. The acclimated microbial consortium originated from a biofilter treating waste gases released from a landfill leachate treatment plant. H_2_S was found as a predominant pollutant in the waste gas with an average concentration of 224 mg/m^3^. Enrichment of the consortium was performed by series of transfer at one-week intervals with thiosulfate as the sole sulfur source. The activated sludge was collected from Liede municipal wastewater treatment plant in Guangzhou, China. The collection of the activated sludge was permitted for research purposes. After settling for 24 h, the concentrated sludge (5 mL) was mixed with the acclimated consortium of 35mL, and added to the nutrient solution (1 L).

### Analytical methods

The pH of the nutrient solution was measured on-line using a pH-meter. To determine the pH of the different layers of the BTFs bed, before each transient experiment, triplicate samples of the packing were taken from the each sampling site using a sterile tweezers, mixed with 3 mL of sterile water, and centrifuged at 7000 rpm for 10 min; the supernatants were analyzed with the pH-meter, and the pellets were used to measure the biomass. The biomass concentration was quantified based on the total protein measurement using the Bradford method. Sulfate, thiosulfate, nitrate and nitrite were analyzed using an ion chromatograph (Dionex ICS-1500, USA) with an AS19/AG19 column (Dionex, USA).

To analyze the H_2_S concentration, gas samples were collected in 2 L Tedlar bags, and immediately analyzed with a gas chromatograph (Shimadzu GC-2010, Japan) equipped with an FPD detector. The air was separated by a GS-GasPro capillary column (30 m × 0.32 mm × 1.0 μm, Agilent Technologies, USA) with nitrogen as the carrier gas at a flow rate of 1.5 mL/min. The injector and detector temperature were 70°C and 250°C, respectively. The GC oven temperature was programmed as follows: initial temperature of 80°C for 2 min, increase to 250°C at 10°C min^−1^ and maintain for 5 min.

### MiSeq sequencing of 16S rRNA gene amplicons

After the acclimation phase, triplicate packing materials of 10 g wet weight were sterilely collected from each layer of both BTFs. The materials were mixed with 100 mL of phosphate buffer (137 mM NaCl, 2.7 mM KCl, 4.3 mM Na_2_HPO4, 1.4 mM KH_2_PO_4_; pH 7.3) and vortexed for 30 min. After detachment, the packing materials were discarded, and the biofilm-containing liquid phase was centrifuged at 8000 rpm for 10min. The resulting pellet was used to extract genomic DNA via the TIANamp Bacteria DNA Kit (Tiangen Biotech, Beijing) according to the manfacturer’s instructions. The DNA density and quality were checked using a NanoDrop Spectrophotometer. The extracted DNA was diluted to 10 ng/μL and stored at -40°C for downstream use. The universal primers 515F(5'-GTGCCAGCMGCCGCGGTAA-3') and 806R(5'-GGACTACHVGGGTWTCTAAT-3') with Illumina barcodes were used to amplify the V4 hypervariable regions of the 16S rRNA genes for pyrosequencing using a Miseq sequencer [29,30]. The PCR mixture (50 μl) contained 1 X PCR buffer, 1.5 mM MgCl_2_, each deoxynucleoside triphosphate at 0.4 μM, each primer at 1.0 μM, 1 U of TransStart Fast Pfu DNA Polymerase (TransGen, China) and 10 ng of soil genomic DNA. The PCR amplification program included initial denaturation at 94°C for 3 min, followed by 30 cycles of 94°C for 40 s, 56°C for 60 s, and 72°C for 60 s, and a final extension at 72°C for 10 min. The PCR products were subjected to electrophoresis using 1.0% agarose gel. The band with the correct size was excised and purified using Gel Extraction Kit (Omega Bio-tek, USA) and quantified with the Nanodrop. All samples were pooled together with equal molar amounts from each sample. The sequencing samples were prepared using the TruSeq DNA kit according to the manufacturer’s instructions. The purified library was diluted, denatured, re-diluted, mixed with PhiX (equal to 30% of the final DNA amount) as described in the Illumina library preparation protocols, and applied to an Illumina Miseq system for sequencing with the Reagent Kit v2 2×250 bp as, following the manufacturer’s instructions.

### High-throughput sequence data analysis

The sequence data were processed using QIIME Pipeline–Version 1.7.0 (http://qiime.org/tutorials/tutorial.html). All sequence reads were trimmed and assigned to each sample based on their barcodes. Multiple steps were required to trim the sequences, such as the removal of sequences < 200 bp and average base quality score Q < 25. The 16S rRNA gene sequences were used for chimera check using the Uchime algorithm. Sequences were clustered into operational taxonomic units (OTUs) using a 97% identity threshold. Each sample was rarefied to the sample exhibiting the lowest number of reads (23,913 sequences) for both alpha-diversity (observed species, Shannon’s and Simpson’s diversity index) and beta-diversity (PCoA, UniFrac) analyses, for which the rarefaction curves were generated from the observed species. Taxonomy was assigned using the Ribosomal Database Project classifier at a confidence level of 80%. OTUs with the abundances exceeding 1% were selected to compare the bacterial communities of the BTFs, and the heat map was plotted using R package.

## Results and Discussion

### Transient behavior of BTFs in removing H_2_S

At the beginning of this study, both BTFs were acclimated under steady state for a month with empty bed retention times (EBRT) of 60 s at an inlet concentration of 607 mg/m^3^. A short time was required to finish the acclimation for both BTFs. Their H_2_S removal efficiencies (REs) rapidly increased to nearly 100% within a week, and maintained this level throughout the rest of the acclimation period. The microbial consortium obtained from a previous biofilter used to treat waste gases from a landfill could be partly responsible for this successful and rapid acclimation.

**Fig 2:**
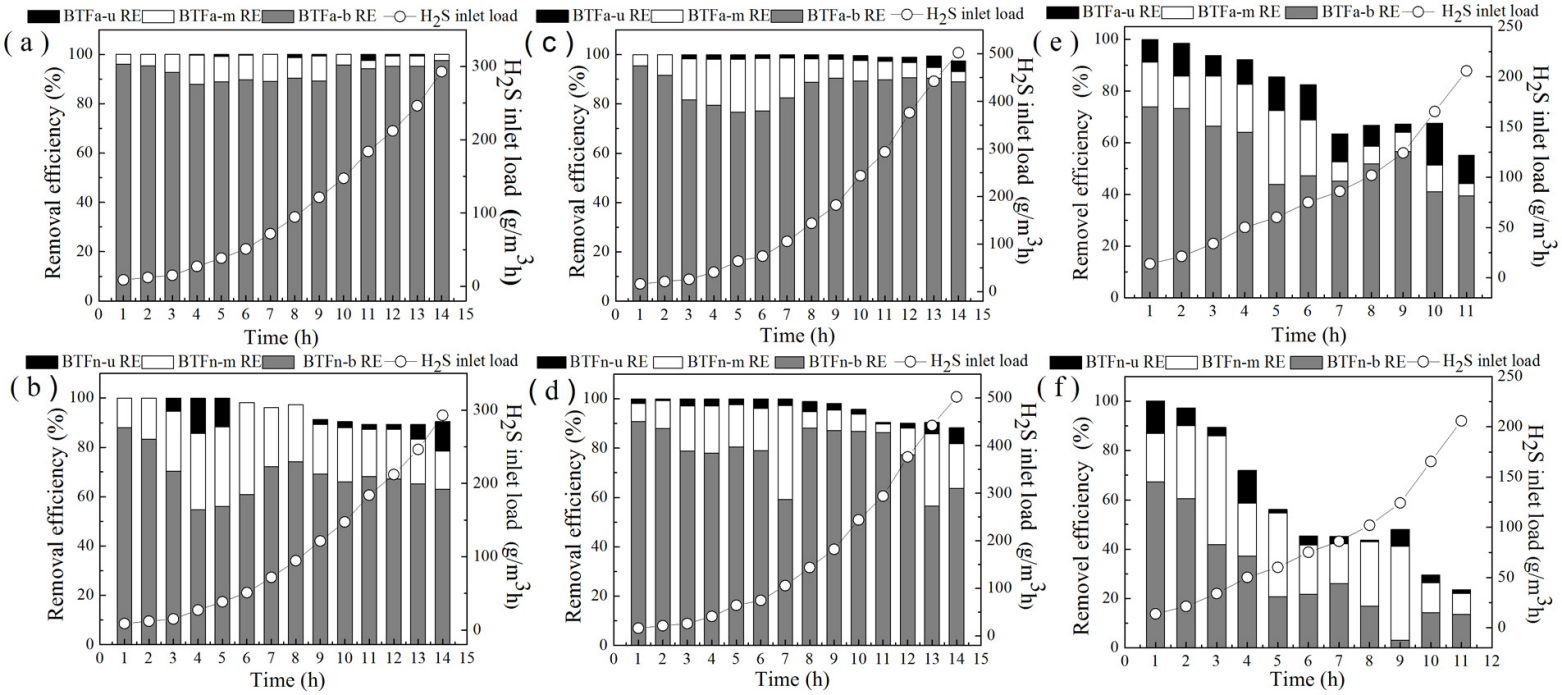
H_2_S removal profile along the column of the two BTFs at the each inlet loading under the EBRTs. (a): EBRT = 60s BTFa; (b): EBRT = 60s BTFn; (c): EBRT = 30s BTFa; (d): EBRT = 30s BTFn; (e): EBRT = 15s BTFa; (f): EBRT = 15s BTFn.

The removal performances of the two BTFs under transient condition were provided in Fig 2. The performances of both BTFs were also evaluated in terms of the removal capacity (EC) at various H_2_S inlet loading rates. The results revealed that an obvious effect of EBRT on the robustness of the BTFs. At an EBRT of 60 s, no negative effect of inlet concentration on the H_2_S REs of BTFa was observed. Indeed, the REs achieved by BTFa at a 60 s of EBRT steadily maintained at near 100% even though the inlet concentration increased drastically from 175 to 5858 mg/m^3^ over 14 h, indicating that BTFa has substantial removal capability at longer EBRTs. The maximum EC at the 60s of EBRT could not be easily achieved because the EC increased linearly when the inlet loading rates ranged from 8.8 to 292.9 g/m^3^.h, and an inlet concentration higher than 5858 mg/m^3^ could not be generated in the present study.

Similar removal behavior exhibited by BTFa was observed at 30s of EBRT with one exception occurred at the highest inlet concentration, but the H_2_S REs only declined by 2.5%. However, when the EBRT was set to 15 s, the H_2_S REs of BTFa dramatically decreased from near 100% to 55.1% while the inlet concentration increased from 69 to 1029 mg/m^3^. These findings were consistent with those of Chaiprapat et al. who reported that H_2_S RE increased 1.44 times in a single stage BTF treating a 5522-ppm H_2_S when the EBRT increased from 100 to 180 s [31]. The maximum EC at 30 and 15s of EBRTs achieved by BTFa were 489.9 and 113.4 g/m^3^ h with inlet loading rates of 502.3 and 205.8 g/m^3^.h, respectively.

The removal capacity of BTFn was decreased as compared with BTFa. The H_2_S REs of BTFn declined while the inlet concentration increased at every EBRT. The extent of the decrease in the REs was more serious than that observed in BTFa, and was also found to depend on the EBRTs employed. Therefore, it can be concluded that BTFa was more robust than BTFn under transient conditions. The maximum EC achieved by BTFn at 60 s, 30 s and 15 s were 265.0, 443.6 and 59.6 g/m^3^.h with inlet loading rates of 292.9, 502.3 and 124.2 g/m^3^.h, respectively. These EC values were significantly lower than those of BTFa. Interestingly, BTFn performed better at 30s of EBRT than at 60s. A previous study suggested that when the EBRT decreases, the H_2_S-removal rates of BTFs were usually limited by the diffusion rate of H_2_S from the gas into the liquid phase rather than by the rate of bacterial consumption of H_2_S [32], since the microorganisms were able to metabolize H_2_S within 1-2s [33]. However, other literature has indicated that as the gas flow rate increases, the gas was forced to flow through the smaller pores, thereby increasing the mobile gas-filled porosity [34].

Fig 2 also provided the profiles of the REs of both BTFs along the filter bed. It can be found that the H_2_S removal mainly occurred in the bottom layer of the filter bed. When the EBRTs exceeded 30 s, average 87.0% and 72.0% of H_2_S were removed in the bottom layer filter of BTFa and BTFn, respectively. In contrast, the upper layers of both BTFs made only a minor contribution to the H_2_S removal (<4.0%). These findings were consistent with those of Montebello et al.,who also reported that most of the H_2_S was removed in the bottom layer, where acidification was more serious due to near the inlet [31,35]. When the EBRTs were set at 15 s, the H_2_S removal contributed to the bottom layer of filter accounted for 69.3% and 47.4% of the total removal in BTFa and BTFn, respectively, indicating that the removal capacity of the bottom layer of BTFa was higher than that of BTFn. The overall removal performance of BTF was generally controlled by either the rate of mass transfer across the gas-liquid interface or the microbial degradation rate [36]. As reported previously, the solubility of H_2_S and O_2_ in acidic conditions was lower than that in neutral or alkaline conditions, thus the H_2_S removal was easily limited by the mass transfer of both H_2_S and O_2_ in acidic conditions [10,18]. In this work, although the initial pH of the nutrient solution added to BTFa and BTFn were always adjusted to 4.0 and 7.0 using an online pH meters and a dosing pump, an obvious pH gradient still formed along the filter bed. The average pH values in the upper, middle and bottom layer of the BTFa and BTFn were 4.04, 2.79, 1.83 and 7.19, 4.97, 2.03, respectively, which means that the overall mass transfer resistance in BTFa should be higher than that in BTFn. In addition, the average biomass in the bottom, middle and uppper layers of BTFa were 2.75, 1.68, 1.47 mg _protein_/g _packing_, which were lower than the corresponding values in BTFn(3.52, 2.43, 2.50 mg _protein_/g _packing_). Therefore, it could be inferred that the microbial degradation rates in most cases was the most important limiting factor of the H_2_S removal in BTFn. However, the specific bio-degrading activity commonly depends on the species of microbe and the growth conditions. Divers microorganisms were supposed to present in both BTFs due to the relative wide range of pH and the vertical stratification of the nutrient and the H_2_S concentration along the filter bed. Thus it was necessary to analyze the structure of the microbial communities for a better understanding the difference in removal behavior of both BTFs.

### H_2_S mineralization

**Fig 3:**
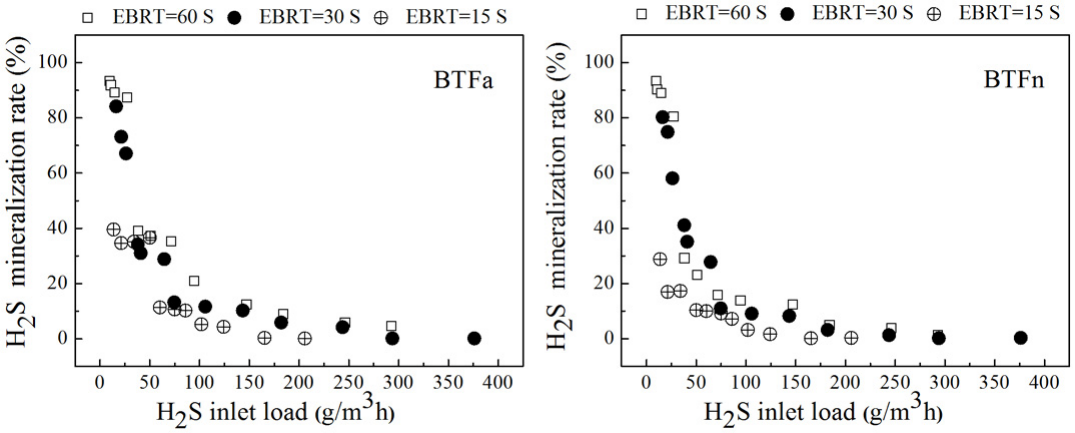
Complete mineralization rate of H_2_S for different EBRTs as the inlet load increases.

The H_2_S mineralization rates were calculated based on the inlet loading rates of H_2_S and the amount of sulfate that accumulated in the leachate. Fig 3 showed that the mineralization rates of H_2_S in both BTFs sharply dropped as the inlet loading rates increased, regardless of the EBRTs tested. For example, as the inlet loading rates increased from 9.6 to 292.9 g/m^3^.h, the rates of H_2_S mineralization at EBRT of 60s in BTFa and the BTFn dropped from 93.3% and 94.4% to 4.5% and 1.3%, respectively. Because that the REs obtained by both BTFs at the EBRT of 60 s were at least 80%, it can be concluded that most of H_2_S were in fact converted into intermediates rather than sulfate. Similar results have been reported in the literature [37]. When the EBRT was set at 15 s, the highest H_2_S mineralization rates were 39.5% in the BTFa and 28.8% in the BTFn, respectively, even when the H_2_S inlet loading rate was only 13.8 g/m^3^.h. The elemental sulfur was detected to accumulate throughout the filter bed, however, it was very difficult to accurately quantify the elemental sulfur due to their uneven distribution in the filter bed. No other S species were detected in the liquid phase, hence the element sulfur can be considered to be the main intermediate of the incomplete bio-oxidation of H_2_S. The accumulation of element sulfur with the input H_2_S loading rates confirmed that the mass-transfer rate was the limiting factor for H_2_S removal. It can also be speculated that the H_2_S removals were mainly limited by the mass-transfer rate of O_2_ rather than that of H_2_S because that the solubility of O_2_ in water was 80 times less than that of H_2_S, and sufficient O_2_ supply typically allows the complete oxidation of H_2_S into sulfate. Rodriguez et al. found that the implementation of an aeration system based on jet-venturi devices results in better conversion of H_2_S to sulfate [38]. Additionally, Chaiprapat et al. suggested that the O_2_/H_2_S ratio was a key factor that controls the level of H_2_S oxidation, and they proposed a three-stage acidic BTF in which aerated recirculating liquid was sprayed on each of the three stages to enhance the oxygen transfer from air to water [31].

### Comparison of bacterial community structure

In this study, the bacterial community structures of the three layers (upper, middle and bottom) of both BTFs were analyzed. High-throughput sequencing of 18 samples yielded 33,557–102,326 sequence reads with an average length of 200 bps. The *β*-diversity of the bacterial communities was represented by the Shannon index. The diversity index values of the upper, middle and bottom layers of BTFa were 8.16, 8.33 and 7.50, whereas those of BTFn were 9.00, 8.48 and 8.25, respectively. These results were inconsistent with those obtained in a previous similar study conducted by Omri et al., which revealed a strong decrease in species diversity towards the upper layer. They concluded that low pH could provide a favorable environment for the growth of sulfur-oxidizing species [16]. This difference may be caused by the methods used in the analysis of the microbial community.

**Fig 4:**
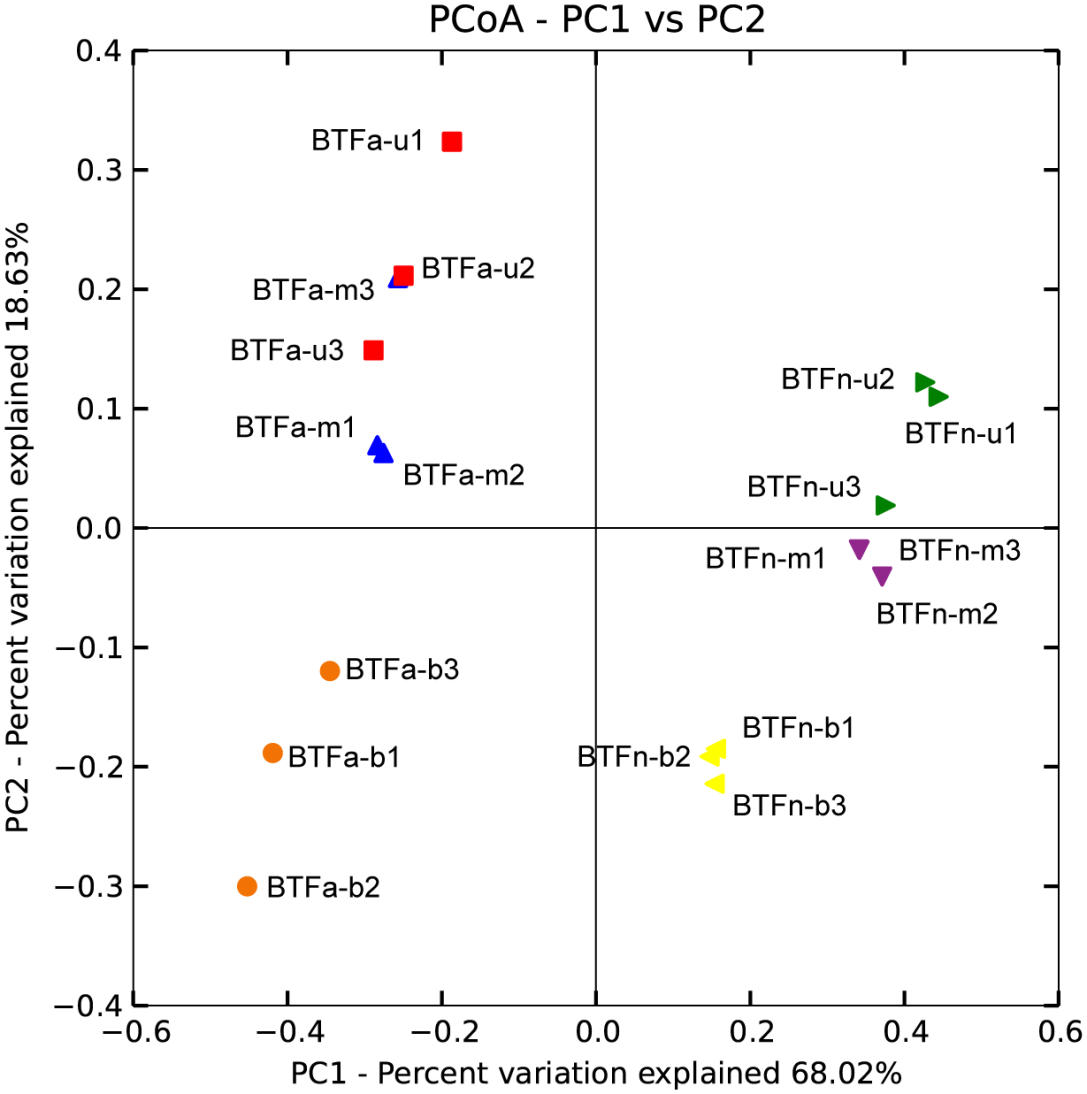
Principal component analysis of the different communities in the tested BTFs.

Principal co-ordinates analysis (PCoA) demonstrated that the data can be reduced to two principal components (PC1 and PC2) with combined Eigenvalues explaining 86.7% of the variation. Fig 4 showed that along the PC1 axis, all samples could be classified into two groups. Samples from BTFa formed one group, which was separate from another group comprised of samples from BTFn. With respect to the vertical distribution, the samples of the bottom layer of each BTF were significantly different from the samples collected from other layers. These results indicated that different bacterial communities occurred in the two BTFs and in different locations of filter bed.

An average of 98.7% of the sequence reads of the individual samples belong to Bacteria and only 1.0% to Archaea by comparing sequence reads to known 16S rRNA genes. Among the Archaea sequence reads, 13.7% and 85.7% were affiliated with the phyla Crenarchaeota and Euryarchaeota, respectively. No significant differences in the relative abundance of the two phyla were found between the BTFs. An average of 92.5% of the Bacteria sequence reads could be classified into 71 different phyla. However, the vertical distributions of the bacterial phyla between the two BTFs were very different.

The relative abundances of the main bacterial phylum were summarized in Table 1. Proteobacteria was the most abundant bacteria phylum in both BTFs with average relative abundances ranging from 56.0% to 77.7%. The percentage of Proteobacteria sequences in the upper, middle and bottom layers of BTFa accounted for 63.9%, 72.6% and 77.7% of the total 16S rRNA gene sequence reads, compared to 56.0%, 66.1% and 66.8% in the corresponding BTFn layers, respectively. Environmental variables involved in this study mainly included the pH, the nutrient and the inlet loading rates of H_2_S. Along the pH gradient in each BTF, the relative abundance of Proteobacteria clearly increased from the upper layer to the bottom layer, correlating with the pH values. However, the abundance of Proteobacteria in similar pH environments very differed between the two BTFs, for example, the pH values in the middle of BTFn and in the upper layer of BTFa were both close to 4.0. Similarly, both pH values in the bottom layers of the two BTFs were near 2.0. These results indicated that other factors also played a role during the evolution of microbial communities. The circulation of the nutrient solution facilitated the shift of microorganisms between layers in the filter bed, therefore resulted in a similar structure of the microbial communities in the same BTF. Additionally, there was second gradient along the column. The H_2_S concentration decreased from the bottom layer to the upper layer. As mentioned above, the removal of H_2_S mainly occurred in the bottom layer which was subjected highest H_2_S loading rates, whereas the concentration of H_2_S in the upper layer gradually decreased to near zero. However, it was not means that the upper and middle layers lacked the sulfur source for the growth of microorganisms. Indeed, the element sulfur that accumulated in the bottom layer due to the incomplete-oxidation of H_2_S, was likely delivered to the upper and middle layers as the nutrients were recirculated.

**Table 1:** The relative abundances of main bacterial phylum.

	Average relative abundance (%)					
	BTFa			BTFn		
Bacterial phylum	BTFa-u	BTFa-m	BTFa-b	BTFn-u	BTFn-m	BTFn-b
*Proteobacteria*	63.9	72.6	77.7	56.0	66.1	66.8
*Alpha proteobacteria*	7.9	4.8	3.0	13.3	14.4	10.8
*Beta proteobacteria*	20.0	32.9	23.6	25.2	29.9	19.1
*Delta proteobacteria*	1.2	1.0	1.2	3.0	2.4	2.9
*Epsilon proteobacteria*	0.0	0.0	0.0	0.0	0.0	0.0
*Gamma proteobacteria*	31.6	29.4	46.7	13.1	18.1	32.7
Other	3.2	4.6	3.1	1.4	1.3	1.3
*Planctomycetes*	8.2	5.2	2.7	8.0	3.2	3.4
*Firmicutes*	6.3	6.2	6.2	5.0	5.6	6.2
*Acidobacteria*	4.6	1.6	1.0	0.9	0.8	0.8
*Actinobacteria*	2.9	2.9	1.2	1.4	1.3	1.4
*Cyanobacteria*	1.0	1.4	0.8	0.2	0.1	0.1
*Bacteroidetes*	1.1	1.1	1.3	10.6	9.5	8.1
*Chloroflexi*	1.0	0.8	0.9	1.0	0.9	1.1
*Verrucomicrobia*	0.4	0.4	0.5	1.8	1.1	1.1
*Gemmatimonadetes*	0.2	0.2	0.2	2.9	1.3	1.6
*Thermi*	0.1	0.1	0.1	3.5	2.3	2.0
Other	6.4	5.0	4.7	4.2	3.6	3.5

The Proteobacteria phylum mainly contained Beta proteobacteria and Gamma proteobacteria classes with average relative abundances of 25.5% and 35.9% in BTFa and 24.7% and 21.3% in BTFn, respectively. Notably, the percentage of Gamma proteobacteria in the upper layer was significantly higher than that in the bottom layer of the filter bed in both BTFs. Alpha proteobacteria was another predominant class in the Proteobacteria phylum. In contrast to the Gamma proteobacteria, the average relative abundance of Alpha proteobacteria in BTFa was significantly lower than that in BTFn. Other predominant phyla included Planctomycetes, Firmicutes, Acidobacteria, Cyanobacteria, Bacteroidetes and Chloroflexi. The spatial distributions of Planctomycetes, Firmicutes and Chloroflexi phyla were similar in both BTFs. However, the spatial distributions of the remaining bacterial phyla were significantly different between BTFa and BTFn.

**Fig 5:**
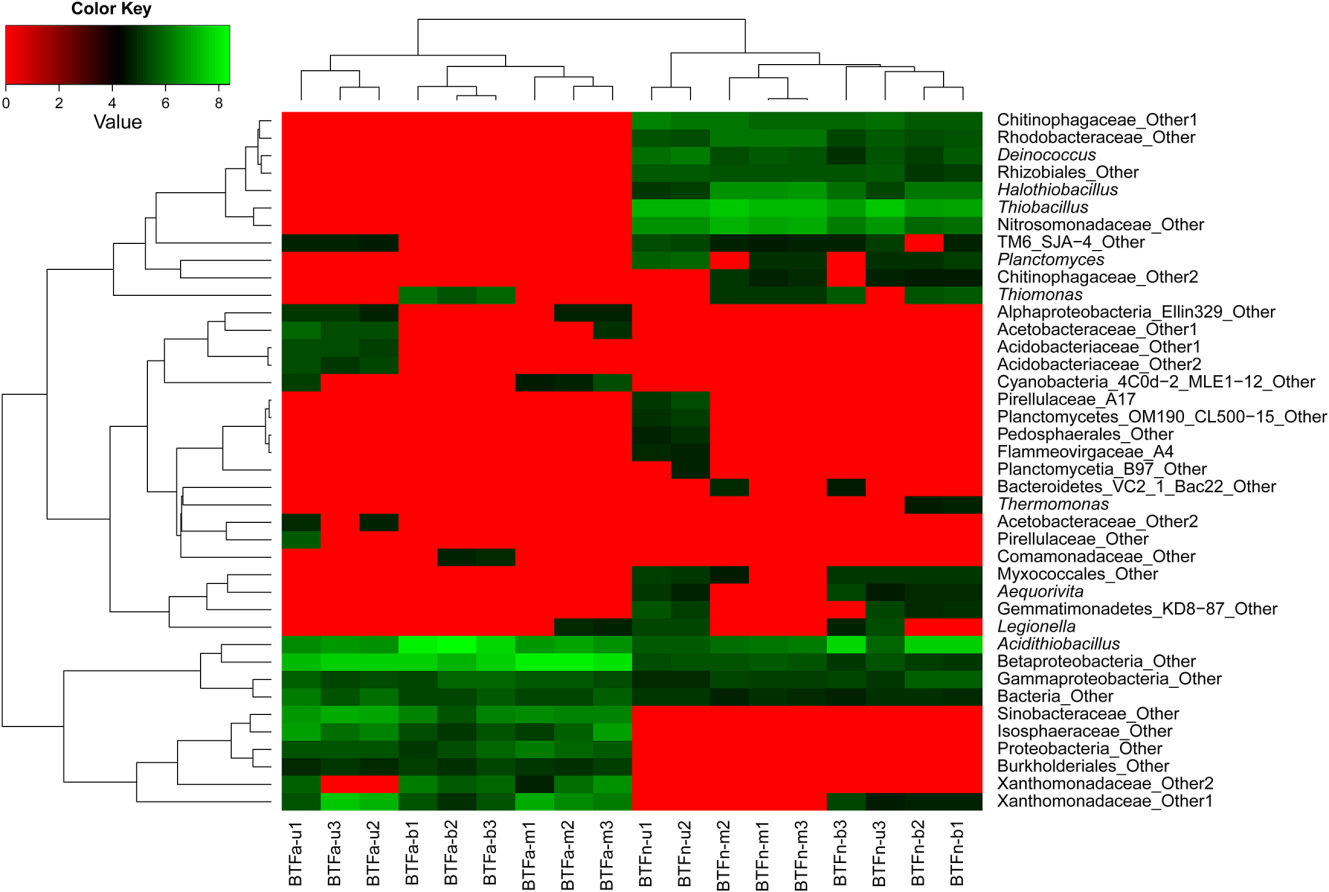
The heat map of the dominant genus in the two BTFs.

When comparing the bacterial community structure at the genus level, differences in relative abundance between BTFa and BTFn increased in significance. As shown by the heat map in Fig 5, the predominant bacterial groups classified at the genus level included *Acidithiobacillus*, *Thiomonas*, *Thiobacillus*, *Halothiobacillus*, *Deinococcus* and *Planctomyces*. Most of these bacterial genera have been reported to have sulfur-oxidizing capabilities [39,40]. In BTFa, *Acidithiobacillus* was the most abundant bacterial group. The proportions of this genus in the upper, middle and bottom layers of BTFa bed were 6.7%, 7.6 and 30.6%, significantly higher than the corresponding values observed in BTFn (2.8%, 4.3% and 18.2%, respectively). Other bacterial groups in BTFa account for less than 1% of the sequences reads. The bacterial species developed in BTFn appear to be more diverse than those in BTFa. The *Thiobacillus* genus was another relatively abundant bacterial group. The vertical distribution of *Thiobacillus* genus in the two BTFs differed from that of the *Acidithiobacillus* genus. The relative abundances of the *Thiobacillus* genus in the upper, middle and bottom layers of BTFn were 12.7%, 13.3% and 8.3%, respectively, whereas the proportion of this genus in BTFa didn’t exceed 0.5%, indicating that *Thiobacillus* was an important H_2_S degrader in higher pH conditions. It were reported that BTFs inoculated with *Thiobacillus thioparus* DW44 or *Thiobacillus thioparus* strains CH11 achieved the high removing efficiency of H_2_S [6,41,42]. The dominant presence of the *Thiobacillus* in the bottom layer of BTFn would also be attributed to the circulation of the nutrient since this genus was reported to be less tolerant to the acidity [19]. The relative abundances of *Halothiobacillus*, *Deinococcus* and *Planctomyces* in BTFn were also significantly higher than those observed in BTFa. pH was confirmed to be a critical factor influencing the formation of the bacterial community structure since the two BTFs were operated under otherwise identical conditions, and received similar H_2_S inlet loading rates. The effects of pH on the formation of the microbial community have been previously reported. Chouari et al. reported that a very acidic biofilter showed a completely different microbial community structure compared to a less acidic biofilter, and with *Acidithiobacillus* dominating in the latter [43].

Although the broader pH gradient formed in BTFn provided diverse environments that facilitated the development of more types of microorganisms in the bioreactor, the sulfur-oxidizing activities of those neutral microbes were easily inhabited under acidic conditions, causing the H_2_S removal to decline. In contrast, the *Acidithiobacillus* genus contains *chemolithotrophic* sulfur-oxidizing bacterium that tend to dominate in extremely acidic conditions [20,44]. Considering the apparently different removal performance exhibited by different reactor and layers, *Acidithiobacillus* in fact played a key role in the H_2_S biofiltration observed in the BTFs, and its abundance of was highly related to the H_2_S removal capabilities of the bioreactors. As mentioned above, the formation of pH gradients along the filter bed were unavoidable even when the pH of the recirculating liquid was readjusted to 7.0 in BTFn and 4.0 in BTFa throughout the experiment. It was very difficult to maintain neutral pH across an entire filter bed. These results indicated that acclimation of the BTF under acidic conditions may facilitate generating a microbial community with high H_2_S-degrading capabilities. The inoculation with *Acidithiobacillus* species was also an ideal alternative to reduce the acclimation times.

## Conclusions

The acidic BTF performed better and robustly than the neutral BTF with regard to H_2_S removal under transient conditions. Considering the different solubility of H_2_S and O_2_, it could be inferred that the microbial activities relating to H_2_S degradation in the BTFa were greater than those in BTFn. H_2_S removal mainly occurred at the down layer of filter bed.

The acclimation under different pH conditions resulted apparently different bacterial communities. Besides pH, the nutrients and the inlet loading also influenced the formation of the bacterial community. The circulation of the nutrient solutions along the filter bed facilitated the transportation of the elemental sulfur and the microorganisms from the bottom layer to upper layer, hence resulted a similar distribution of microorganisms within same BTF. *Acidithiobacillus* genus was the most abundant bacterial group under acidic conditions.
